# Material Model Development of Magnesium Alloy and Its Strength Evaluation

**DOI:** 10.3390/ma14020454

**Published:** 2021-01-19

**Authors:** Wenjia Huang, Ninshu Ma, Yunwu Ma, Toshiro Amaishi, Kenji Takada, Takayuki Hama

**Affiliations:** 1 Joining and Welding Research Institute, Osaka University, Osaka 567-0047, Japan; huang.wj@jwri.osaka-u.ac.jp; 2 Division of Global Architecture, Graduate School of Engineering, Osaka University, Osaka 565-0871, Japan; 3 JSOL Corporation, Osaka 550-0001, Japan; amaishi.toshirou@jsol.co.jp; 4 Honda Motor Co., Ltd., Tokyo 107-8556, Japan; kenji_takada@jp.honda; 5 Division of Resources and Energy, Graduate School of Energy Science, Kyoto University, Kyoto 606-8501, Japan; hama.takayuki.4s@kyoto-u.ac.jp

**Keywords:** material model, magnesium, finite element method, strength analysis, automobile parts

## Abstract

A new material model of magnesium alloys, combining both Hill’48 yield function and Cazacu’06 yield function, was developed and programmed into LS-DYNA using user subroutine, in which both slip dominant and twinning/untwinning dominant hardening phenomena were included. First, a cyclic load test was performed, and its finite element analysis was carried out to verify the new material model. Then, the deformation behaviors of the magnesium crash box subjected to the compressive impact loading were investigated using the developed material model. Compared with the experimental results, the new material model accurately predicted the deformation characteristics of magnesium alloy parts. Additionally, the effect of the thickness distribution, initial deflection and contact friction coefficient in simulation models on deformation behaviors were investigated using this validated material model.

## 1. Introduction

Nowadays, magnesium alloys have been used in transportation, electronics, medical industries due to their good lightweight properties, machinability, corrosion resistance, shock absorption, dimensional stability and impact resistance. For example, in automotive applicants, low-temperature components like brackets, covers, cases of modern automotive are made of magnesium alloys [1,2,3,4,5,6,7]. In biomedical applications, bioimplants, which are devices that replace the affected or damaged part of the human body and assist in the normal functioning of the human body with a high degree of physiological acceptance, were made by magnesium alloys [8,9,10,11]. For example, Amerinatanzi et al. performed a prediction of the biodegradation of magnesium alloy implants [12]. With good application prospects and great potential, magnesium alloys have become one of the hot issues of new materials in the future [13,14].

Numerical simulation is an important procedure for the design and optimization of magnesium alloy structures. The reliability of numerical analysis depends on the accuracy of the material model. Developing a constitutive model of the magnesium alloys, however, is a rather challenging task compared to cubic metals because magnesium alloys have a hexagonal lattice structure, which affects the fundamental properties of these alloys. The hexagonal structure limits easy dislocation motion to the close-packed direction on the basal planes, and plastic deformation of the hexagonal lattice is more complicated than in cubic latticed metals like aluminum, copper and steel. A material model for magnesium alloys should be able to describe these anisotropy, asymmetry and temperature-dependent behaviors [15,16,17,18,19,20,21,22].

For quite some time, many researchers have been working on the simulation of magnesium behavior. Saniee et al. [23] considered the flow behaviors of several magnesium alloys in tension and compression. On the other hand, Agnew et al. [24] validated an elastoplastic self-consistent polycrystal model that was used to simulate the macroscopic flow curves and internal strain developments within the distinctly textured magnesium alloy samples. Staroselsky et al. [25] also applied a constitutive model hexagonal close-packed (HCP) material deforming by slip and twinning to magnesium alloy AZ31B. In the application aspect, Zhu et al. [26] put forward two optimization-based methodologies to calibrate material parameters for the application of AM60B magnesium alloy material model to structure component crush analysis. A three-dimensional finite element model (FEM) was established by Shen et al. [27] to simulate the temperature distribution, flow activity, and deformation of the melt pool of selective laser melting (SLM) AZ91D magnesium alloy powder. Samuha et al. [28] improved formability for the commercial magnesium AZ80 alloy through the application of the high-rate electromagnetic forming (EMF) technique. Ma et al. [29] developed a temperature-dependent anisotropic material model combined with the Hill48 yield function for the warm-drawing of magnesium alloys. A new extrusion process for Mg–Al–Mn–Ca magnesium alloys using rapidly solidified powders produced very fine grains was also developed by Ma et al. [30].

Kim et al. [31] proposed a constitutive model that combined both Cazacu’06 yield criterion [32] and Hill’48 yield criterion [33] to describe the temperature-dependent asymmetric cyclic behavior of magnesium alloy with the concept of the dominant deformation mode twinning (T), untwinning (U) and slip (S) in 2013. Although effective, this theory was complex to be realized in a computation program.

Therefore, this paper develops a new material model facing practical industry application based on Kim’s theory [31] in LS-DYNA. First, we performed a cyclic loading test and employed a one-shell element model to verify the accuracy of the new material model. Then, this new material model was applied to predict the deformation behavior of magnesium alloy made crash box. The simulation results showed good agreement with the experiment. Several factors were investigated, such as wall thickness, friction coefficient and initial deflection, to try to find which factor led to the deviation between the simulation results and the experiment results. It was found that the initial deflection, friction coefficient and thickness of the wall may be the considerable and reasonable factors that led to the error.

## 2. Materials and Methods

**Figure 1:**
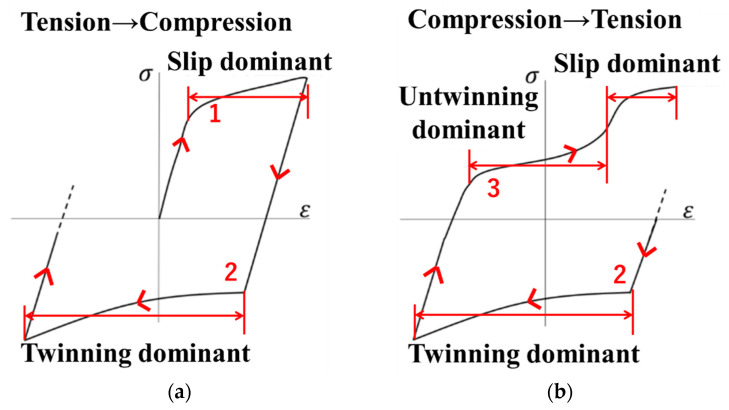
Asymmetry of stress-strain curves: (**a**) tension to compression; (**b**) compression to tension.

To describe the material properties of an HCP metal and alloys, Cazacu et al. proposed a yield function (Cazacu’06) [32] that can capture the asymmetric yield surface of magnesium alloys. The asymmetry of the yield condition of magnesium alloy was caused by deformation based on twinning and untwinning. Schematic stress–strain curves in tension–compression and then compression–tension is shown in Figure 1.

For the accurate simulation of the deformation of the HCP material, considering the effect of the twinning was important. Kim et al. [31] introduced an idea that considered three modes as twinning, untwinning and slip, to describe the cyclic loading behavior of magnesium alloys by using the combination of the Hill’48 [33] and Cazacu’06 [32] yield functions. To conduct a finite elment analysis (FEA) simulation based on the constitutive model, which considered twinning, untwinning and slip modes, the introduced model was implemented to commercial FEA code LS-DYNA by using a user material subroutine. The new material model focused more on the practical application. Hence, some parts of Kim’s model were simplified to reach high-efficiency with acceptable accuracy. The Hill’48 [33] yield function was used for describing the slip-dominant deformation. For simplification, plane stress conditions and only transverse anisotropy were assumed. Then, its effective stress was given by:

(1) $${\bar{\sigma }}_{S}=\sqrt{\sigma_{11}^{2}+\sigma_{22}^{2}-\left(\frac{2r}{1+r}\right)\sigma_{11}\sigma_{22}+2\left(\frac{1+2r}{1+r}\right)\sigma_{12}\sigma_{21}}$$

where r was the average Lankford value. $\sigma_{11}$, $\sigma_{22}$, $\sigma_{12}$ and $\sigma_{21}$ were normal components of the Cauchy stress in the loading direction. The slip deformation was dominant in the tensile-loading condition without twinning history. On the other hand, the twinning deformation was dominant in the compression loading condition. Cazacu’06 [32] yield function was used for the twinning and untwinning dominant deformation. Assuming the plane stress condition and planar isotropy; then, effective stress is given by:

(2) $${\bar{\sigma }}_{t}=\frac{\beta +1}{2\beta }{\left[{\left\{\left|\sigma_{1}\right|-\left(\frac{\beta -1}{\beta +1}\right)\sigma_{1}\right\}}^{a}+{\left\{\left|\sigma_{2}\right|-\left(\frac{\beta -1}{\beta +1}\right)\sigma_{2}\right\}}^{a}\right]}^{\frac{1}{a}}$$

where a was the exponent of the yield function, and β was the parameter of stress ratio. $\sigma_{1}$ and $\sigma_{2}$ were the in-plane principal values of the Cauchy stress. In this study, exponent *a* was set 2.0. β was the parameter of the stress ratio, which was defined by using compressive yield stress ${\hat{\sigma }}_{t}$ and tensile yield stress ${\hat{\sigma }}_{u}$ for the twinning and untwinning dominant deformation in Equation (3):

(3) $$\beta =\frac{{\hat{\sigma }}_{u}}{{\hat{\sigma }}_{t}}$$

During the tensile deformation with twinning history, if the following condition Equation (4) was satisfied, the constitutive equation was computed as untwinning deformation was dominant.

(4) $$C{\bar{\varepsilon }}_{t}^{p}-{\bar{\varepsilon }}_{u}^{p}>0$$

where *C* was the scale value, ${\bar{\varepsilon }}_{t}^{p}$ was effective plastic strain by twinning deformation and ${\bar{\varepsilon }}_{u}^{p}$ was effective plastic strain by untwinning deformation.

**Figure 2:**
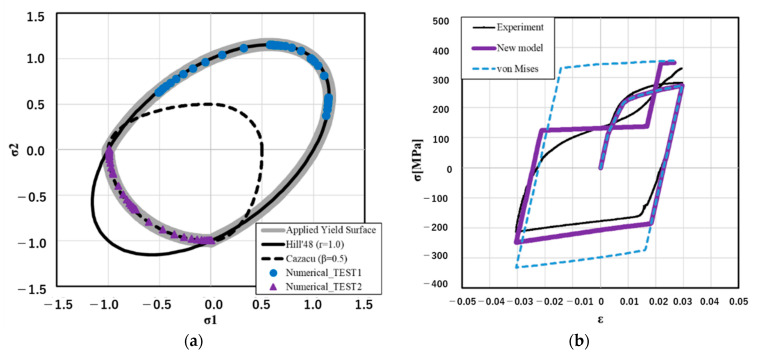
Validation of the newly implemented material model: (**a**) normalized yield surface; (**b**) cyclic stress-strain history.

Then, how the new material model works in the plane stress state is shown in Figure 2a. The thick gray line shows the normalized yield surface used in this research, which is the combination of the Hill’48 [33] and the Cazacu‘06 [32] yield functions. Numerical_TEST1 and Numerical_TEST2 were the verification points at different stress ratios.

Let $\sigma_{m}$ be the mean stress defined as below:

(5) $$\sigma_{m}=\left(\sigma_{11}+\sigma_{22}+\sigma_{33}\right)/3$$

When the tension, $\sigma_{m}\geq 0$ and $C{\bar{\varepsilon }}_{t}^{p}-{\bar{\varepsilon }}_{u}^{p}\leq 0$, yield stress was under slip control, the Hill’48 [33] yield function was used for elastic-plastic calculation. When compression, $\sigma_{m}<0$, yield stress was controlled by twinning, and the Cazacu’06 [32] yield function was used for elastic-plastic calculation. When tension, $\sigma_{m}\geq 0$ and $C{\bar{\varepsilon }}_{t}^{p}-{\bar{\varepsilon }}_{u}^{p}>0$, yield stress was controlled by untwinning, so in the elastic-plastic calculation, the Cazacu’06 yield function could be used.

As an example of the validation of the new material model, tension–compression–tension loading tests using AZ91 and numerical calculations were carried out. Their results are shown in Figure 2b. The strain was set as 0% → +3% → −3% → +3% and strain rate was 0.1. For the comparison, the calculation result of the fundamental elastic-plastic model (von Mises, V-M) is also included in Figure 2b. The new material model considered two stress–strain curves; one was a stress–strain curve for slip deformation, the other one was a stress–strain curve for twinning deformation as input data. The yield stress during untwinning dominant deformation was described by using yield-stress based on twinning and the stress ratio parameter *β*. This result was consistent with the result given by Kim’s model. In this study, parameter *β* was treated as a constant value for the simplification. Specifically, parameter *β* was set as 0.5 in the validation example; how this was determined is introduced in Section 4. When the stress ratio *β* could be defined as nonconstant data, the relation between twinning and untwinning dominant yield stress could be reproduced more flexibly.

## 3. Experiments

**Figure 3:**
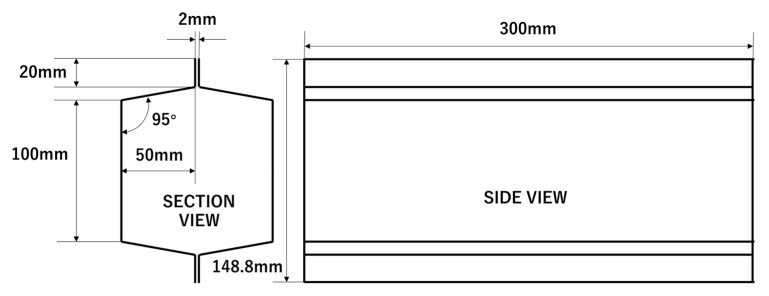
Dimensions of crash box.

The research object was a crash box, which was an energy-absorbing device installed in order to reduce the repair costs in low-speed vehicle collisions. The overall size of the crash box is shown in Figure 3. The nominal wall thickness was 2.00 mm.

**Figure 4:**
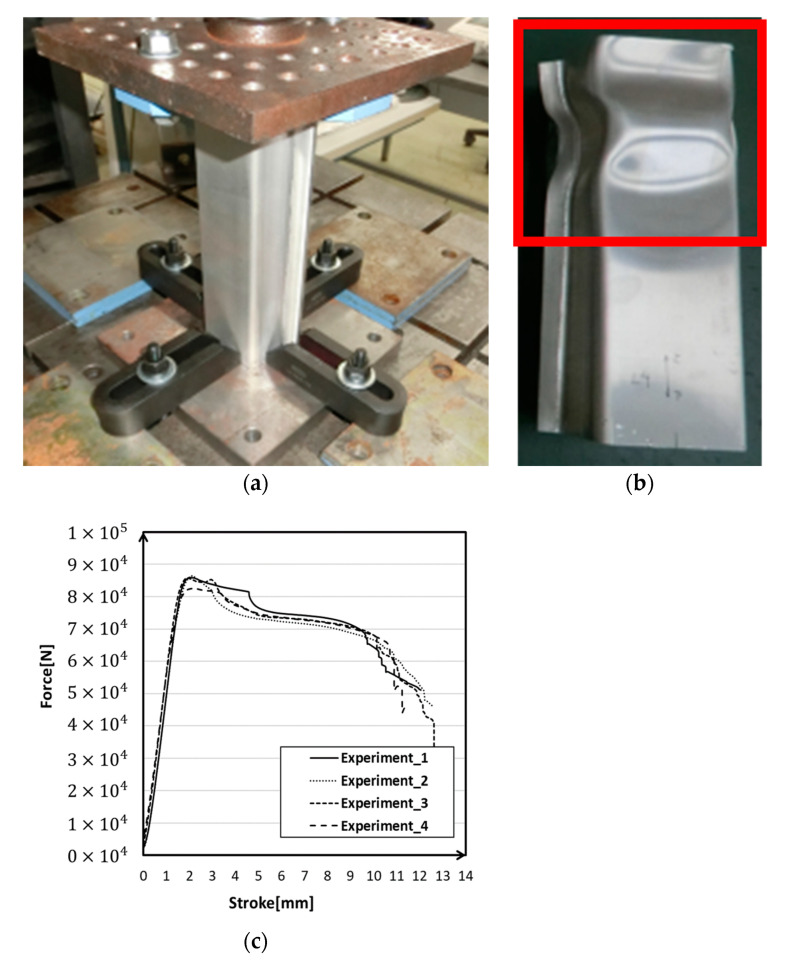
Experiment device and results: (**a**) loading device; (**b**) deformed crash box; (**c**) reaction force verse stroke curves.

The experimental setup of the impact load experiment is shown in Figure 4a. An autograph of 100 kN was used, and the loading speed was 10 mm/min. The bottom of the frame was fixed to prevent lateral movement, and the top of the frame was free. Finally, reaction forces and stroke data were measured from the autograph. Figure 4b gives the deformation mode of the experiment, and Figure 4c plots the experiment data of reaction force versus stroke. The impact tests were repeated four times. All of the crashed samples showed similar features, so we chose the most representative one.

## 4. Numerical Analysis

### 4.1. Mesh and Boundary Condition

**Figure 5:**
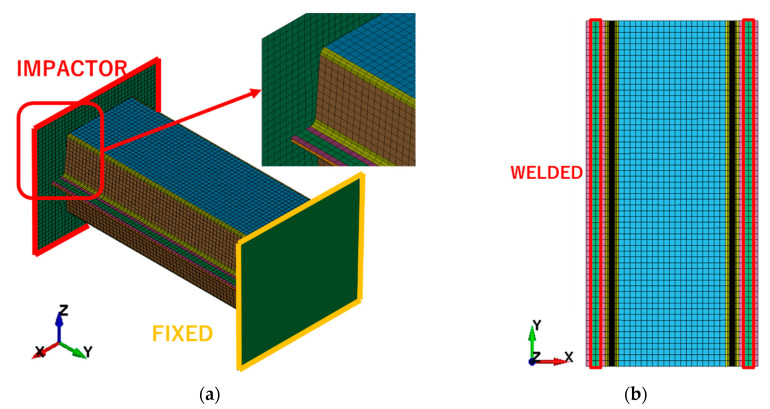
FEA model of the crash box: (**a**) Isometric view; (**b**) front view.

The FE model consisted of 7906 shell elements with a thickness of 2.00 mm. Two main parts of the crash box model were connected by the welded flanges (the green parts), while the welding strength was not considered in this model (Figure 5). The bottom nodes of the model were fixed to a rigid surface. The impact load in the Y direction was applied by a shell surface moving in the Y direction with a constant speed, which equaled 100 mm/s. The dynamic and statistical friction coefficient between the shell surface and the box were both 0.1.

### 4.2. Thickness Measurement

**Figure 6:**
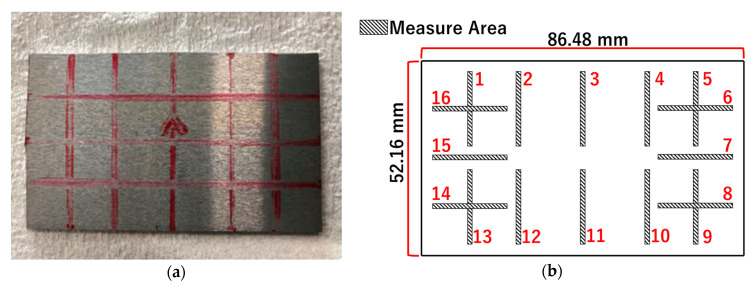
Thickness measurement: (**a**) physical image of the test piece; (**b**) locations of the measured points.

To measure the wall thickness used in this structure, one small test piece, measuring 86.48 mm × 52.16 mm, was cut from the crash box, and the thickness of the test piece was measured by Vernier calipers. Finally, we got 16 sets of data. Figure 6 is a diagram of the measured area.

The measured thickness data at different locations are summarized in Table 1.

As can be seen from this table, there was some error between the actual size and the design size. According to the calculated average thickness of the test piece, the thickness of shell elements in the finite element model was set to 1.96 mm as true thickness.

### 4.3. Material Properties

In this research, magnesium alloy AZ91 was used, and the material properties are shown in Table 2. *a* was an exponent of the Cazacu’06 yield function, refer to Equation (2), and *β* was yield stress ratio of twining and untwinning, refer to Equation (3). *C* was a scale value that determines the untwinning condition refer to Equation (4).

According to Equations (6) and (7), the bulk module bk and shear module *g* could be calculated by:

(6) $$bk=\frac{E}{3\left(1-2v\right)}$$

(7) $$g=\frac{E}{2\left(1+v\right)}$$

Hence, the next step was to determine the value of parameters β and C in this research. As mentioned in the new material model introduction, β was the ratio of compressive yield stress ${\hat{\sigma }}_{t}$ and tensile yield stress ${\hat{\sigma }}_{u}$. Intuitively, β determined the first turning point in the process of changing from compression to tension, which presents the twinning-dominant turning to untwinning-dominant in the stress–strain curve. C determined the second turning point in the process of changing from compressive strain to tensile strain. Three different values of β and C are given in Table 3.

For the simulation, we created a 10 mm × 10 mm square shell element and gave it a one-direction loop displacement from −0.3 mm to 0.3 mm. The loading condition was tensile, compression, then tensile. The order was different from the experiment because the input data of the tensile-compression curve in the simulation was measured in the process of compression first.

**Figure 7:**
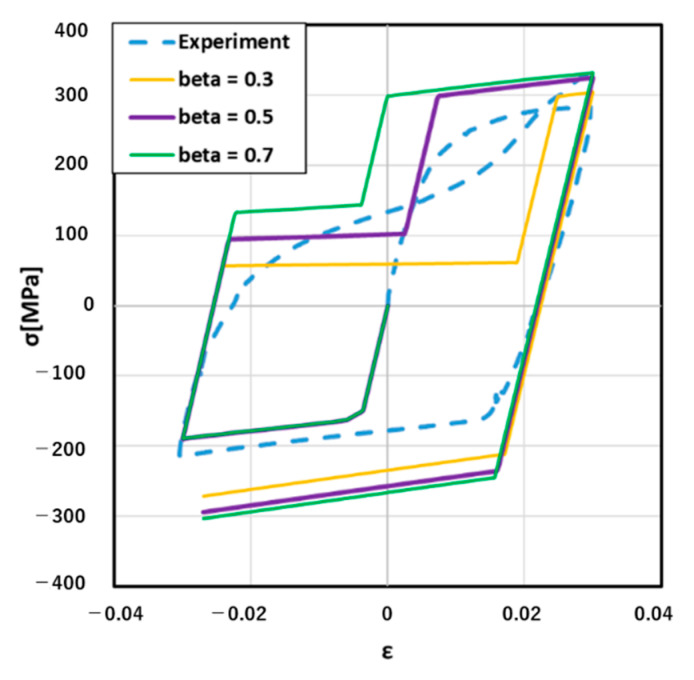
Results with different values for β.

**Figure 8:**
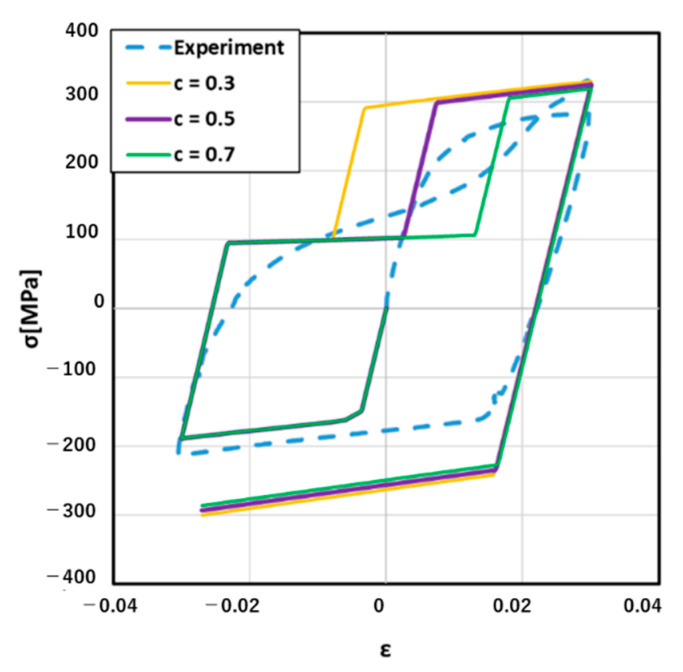
Results with different values for C.

Figure 7 clearly shows how could β affect the stress–strain curve result. For example, when β equaled to 0.5, untwinning dominant tension stress was 100 MPa and twinning dominant compression stress was −200 Mpa, which was described in Equation (3). When β equaled to 0.3, untwinning dominant tension stress decreased to 60 MPa, and the range of untwinning dominant became wide. On the contrary, when β was 0.7, untwinning dominant tension stress increased to 140 MPa, and the range of untwinning dominant becomes narrow. Hence, through Figure 7, we could find that when β=0.5, the simulation result was the closest to the experiment stress–strain curve. Hence, it was reasonable to set β as 0.5 in the next step of the simulation. On the other hand, C only affected the region of untwinning dominance. When C was small, the region of the untwinning dominance would be narrow. In Figure 8, the suitable value of C was 0.5.

**Figure 9:**
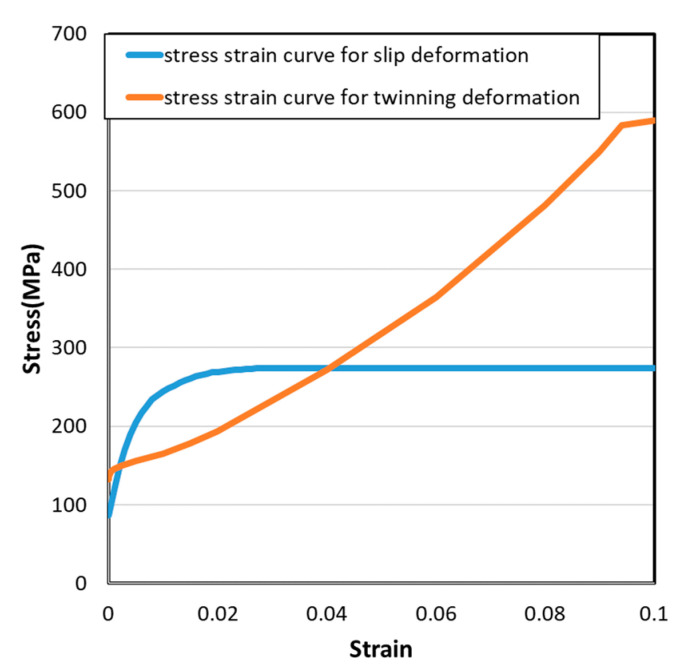
Input stress–strain (SS) curves for slip deformation and twinning deformation.

The stress–strain curve for slip deformation and stress–strain curve for twinning deformation are shown in Figure 9. The stress–strain curve for slip deformation was derived from cyclic loading test results’ tension part. The stress–strain curve for the twinning deformation used compression side stress–strain curves after tension as input data. Based on the slopes of the curves, extrapolated points were included in these curve data.

### 4.4. Simulation Results

**Figure 10:**
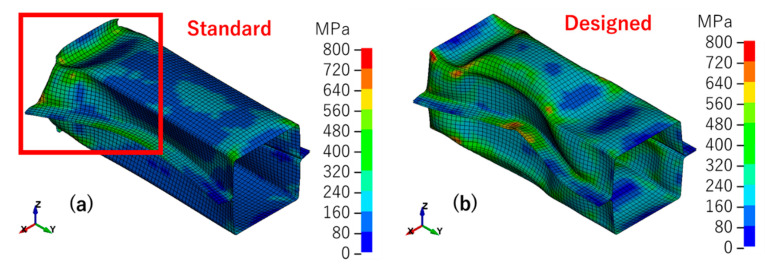
Von Mises effective stress: (**a**) standard model; (**b**) designed model.

**Figure 11:**
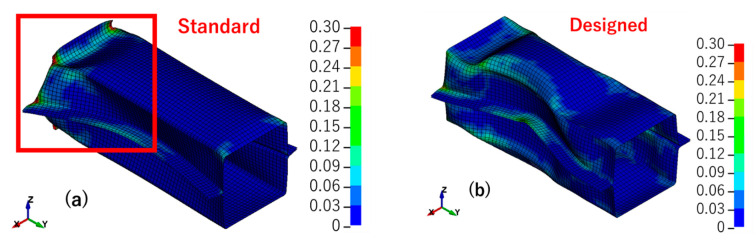
Effective plastic strain: (**a**) standard model; (**b**) designed model.

**Figure 12:**
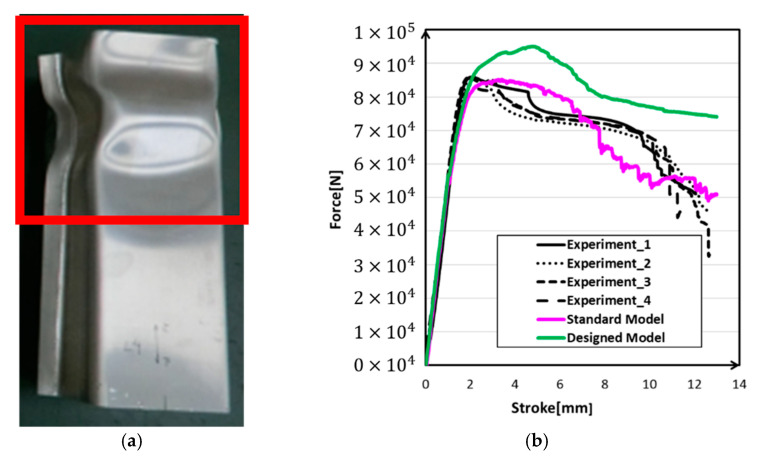
Experiment result: (**a**) deformation mode; (**b**) force-stroke curves.

The results of the simulation were evaluated in terms of deformation mode, von Mises effective stress (Figure 10), effective plastic strain (Figure 11) and reaction force vs. stroke curve (Figure 12). The standard model was the model that was optimized by the real shell thickness (1.96 mm) and proper friction coefficient (0.1), and the designed model was that the one employed designed shell thickness (2.00 mm) and friction coefficient (0.2).

Figure 10 presents the von Mises effective stress results of the standard and designed models. It is clear that the two models had different deformation modes. There was more deformation that appeared at the top of the structure in the standard model, but the designed model showed more like a symmetry wavy deformation. The thinning of the wall thickness led to a decrease in local stiffness, which caused local yield before the load causes the overall yield. With the decrease of stiffness, deformation became easy to happen and also, the stress decrease. Then as shown in Figure 11, large deformation caused large strain, and the standard model’s strain concentration was stronger than the designed model’s strain concentration. Figure 12 shows the experiment deformation mode and reaction force of the two models. The experiment deformation mode was consistent with the standard model’s deformation mode presenting a convex–concave–convex trend. In the elastic period of the reaction force versus the stroke figure, there was no significant difference between the two models; both of them demonstrated a good fit in terms of stiffness of the structure. However, when it came to the plastic period, in the designed model, the stress–strain curve showed a strong work hardening. This brought a maximum reaction force of about 10% higher than the experimental data and the standard model. The standard model had the same level of the maximum reaction force as the experiment. Factors that may affect this result would be discussed in the next section.

## 5. Case Study of Magnesium Material Models and Manufactured Shape Error

When modifying the designed model, we found that there were several factors that could affect the results of the simulation results at the same time. In order to figure out the relationship between these factors and simulation results, we performed the following case studies, as shown in Table 4.

### 5.1. CASE_1: Effect of Shell Thickness

In real industrial production, there are dimensional errors. Therefore, this point needed to be considered in the simulation calculation. In this research, the wall thickness could be an important parameter that could affect the simulation results. In order to understand the effect of plate thickness on simulation results, four models—including designed thickness (a), measured thickness (b), −5% thickness (c) and −10% thickness (d)—were compared in this section. Table 5 presents the detailed information of four models.

**Figure 13:**
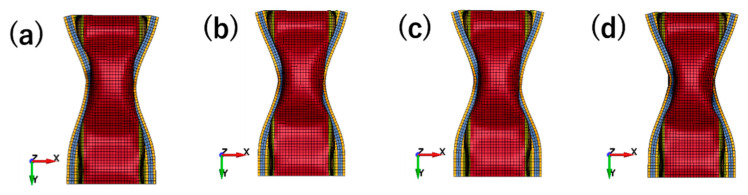
Deformation modes under different wall thickness: (**a**) 2.00 mm; (**b**) 1.96 mm; (**c**) 1.90 mm; (**d**) 1.80 mm.

**Figure 14:**
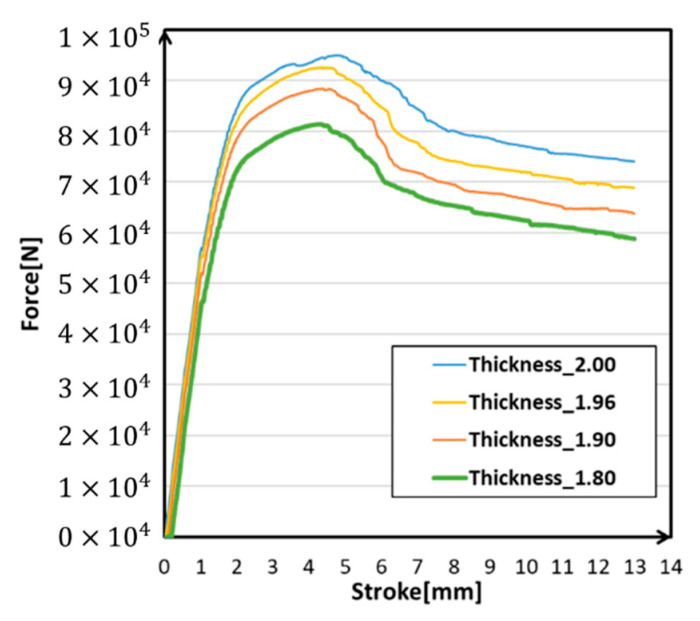
Force-stroke curves under different wall thickness.

Looking into the three models of thickness change, it was easy to find that all of these four models showed the same features in Figure 13. As the wall thickness decreased, the degree of deformation in the middle of the structure increased. It was also notable that changes in the reaction force curve were proportional to changes in thickness and kept the same characters (Figure 14). Although the thickness could affect the simulation results, the work hardening had not been weakened. Hence, the thickness was the main factor that caused the change of strength in the entire structure and kept features of the reaction force at the same time.

### 5.2. CASE_2: Effect of Friction Coefficient

As shown in the photos of low-speed impact load experiments (Figure 4b), there were some very interesting phenomena. In some conditions, a slight slide occurred in the contact surface of the impactor and structure. This reminded us that the contact condition of the impactor and crash box might play important roles in effect simulation results. Hence, we discussed the three conditions of statistic fraction (FS) and a dynamic fraction (FD) in Table 6; These two values were usually given from 0.01 to 0.2.

**Figure 15:**
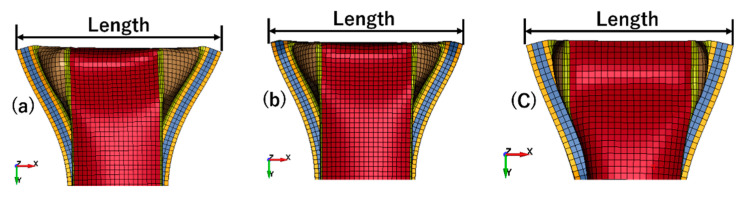
Comparison of slide degree under different friction condition: (**a**) = 0.05; (**b**) = 0.10; (**c**) = 0.20.

**Figure 16:**
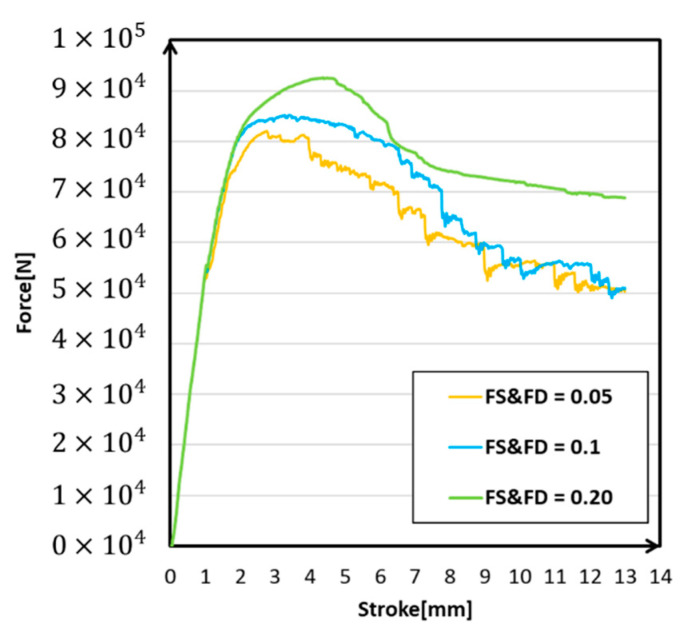
Force-stroke cruves under different friction conditions.

Due to the decrease of friction coefficient, the deformation mode changed. The slide on the contact surface made deformation at the contact part become bigger than before (Figure 15). These models presented the decrease in reaction force, and the smaller the friction coefficient was, the smaller the reaction force in Figure 16. This was because when the friction coefficient decreased, the deformation at the connecting part became larger, which could be concluded by measured top expansion length in Table 7. This made the axial compression shift and then led to the stiffness being weakened. Finally, the reaction force decreased. Model (a) showed a lower value of maximum reaction force; both models performed similarly in tendency. The decrease of friction coefficient changed the deformation mode locally and decreased the maximum reaction force as well. In such a contact condition, 0.1 was a more reasonable value than 0.05 when setting the friction condition.

### 5.3. CASE_3: Effect of Initial Deflection

**Figure 17:**
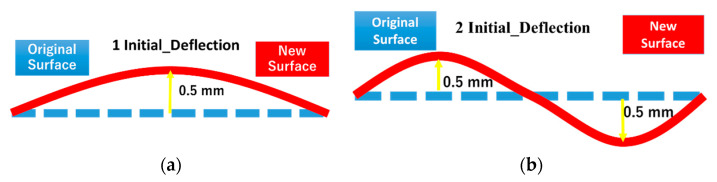
Two types of initial deflection: (**a**) arched type; (**b**) wavy type.

In addition to the above factors, we speculated that the initial deflection might also affect the stiffness of the structure, according to the former experience. It can also be another reason for the slip, as same as the friction coefficient. To figure this out, two types of initial deflection types were put forward, and the deformation at the peak was up to 0.5 mm, which was considered to be an acceptable error in engineering, as shown in Figure 17. Type 1 was a arched type. Type 2 was a wavy type. Other deformation values of points in the surface were fitted by a linear function.

**Figure 18:**
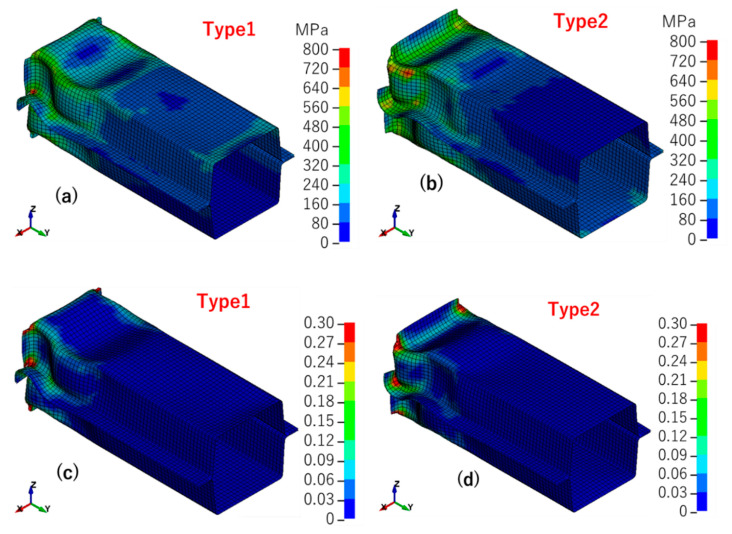
Simulation results: (**a**) Von-Mises (V-M) effective stress of type 1; (**b**) effective plastic strain of type 1; (**c**) V-M effective stress of type 2; (**d**) effective plastic strain of type 2.

**Figure 19:**
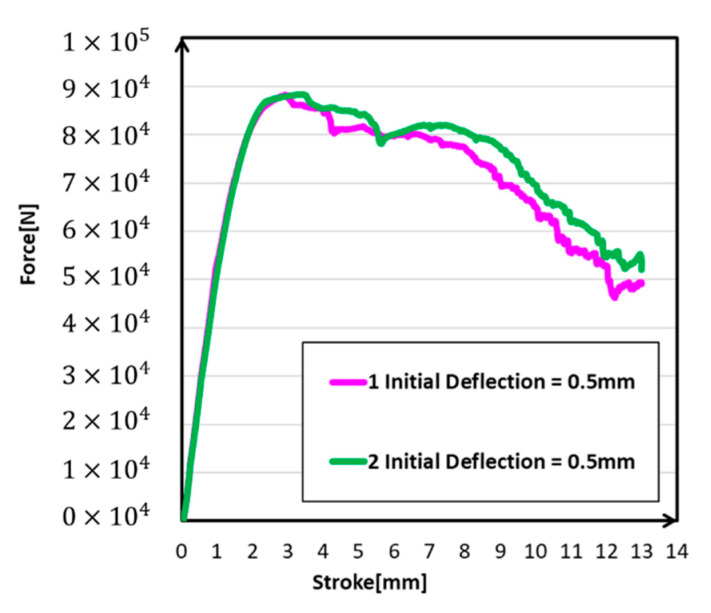
Force-stroke curves under different types.

Different from the designed model, the deformation of the initial deflection model was concentrated in the upper part of the structure. This may because that the initial deflection made the upper part become easier to bend, and instability appeared earlier. Deformation was also much stronger at the contact part in initial deflection models than in the standard model (Figure 18). The deformation characteristics of the two models were a little bit different from each other; the deformation peak of the wavy type was closer to the contact part. This was because the peak of initial deflection in wavy type was more than that in arc type. As shown in Figure 19, there was little difference between the tendency of the two initial deflection types, except the appearance of the maximum reaction force was a little bit earlier in arc type. However, the maximum reaction force of the two types was almost at the same level. This was because the wavy type made the model more prone to instability and earlier to reach the maximum reaction force. According to the comparison, the initial deflection should be a considerable and reasonable factor that affected the simulation results.

## 6. Conclusions

Following the comparison of the traditional material model and investigations of the influence on impact simulations that may be caused by several factors such as wall thickness, friction coefficient and initial deflection, the following conclusions can be drawn:

♦ The new material model based on a combination of the Hill’48 yield function and the Cazacu’06 yield function indeed had a better ability to describe the behavior of magnesium alloy material and also raised the accuracy of impact load simulation results;
♦ The simplification in the new material model by using parameter *β* and C could satisfy the accuracy need in practical industry application;
♦ The increase of the wall thickness results in the increased reaction force, however, has little influence on the deformation mode.
♦ The friction coefficient and initial deflection have significant influence on the local strength of the structure and the deformation mode.
♦ All these three factors should be considered in simulating deformation behaviours of practical parts under impact loading.

## Figures and Tables

**Table 1:** Thickness measurement data.

No.	Thickness (mm)	No.	Thickness (mm)
1	1.97	9	1.95
2	1.96	10	1.96
3	1.96	11	1.97
4	1.97	12	1.97
5	1.97	13	1.97
6	1.94	14	1.95
7	1.94	15	1.95
8	1.94	16	1.96
Average Thickness (mm)		1.96	

**Table 2:** Material properties.

Density (g/mm^3^)	Elastic Modulus (MPa)	Poisson’s Ratio	Bulk Modulus (MPa)	Shear Modulus (MPa)
$1.8\times 10^{-3}$	41,000	0.3	34,167	15,769
a	*β*	C		
2.0	0.5	0.5		

**Table 3:** Values for beta and C.

β	C
0.3	0.5
0.5	0.5
0.7	0.5
0.5	0.3
0.5	0.5
0.5	0.7

**Table 4:** Summary of the case study parameters.

Case	Effect Factor	Thickness (mm)	Friction Coefficient	Initial Deflection
1	Thickness	(a) 2.00	0.20	none
		(b) 1.96		
		(c) 1.90		
		(d) 1.80		
2	Friction coefficient	1.96	(a) 0.05	none
			(b) 0.10	
			(c) 0.20	
3	Initial deflection	1.96	0.20	Type1
				Type2

**Table 5:** Models with different wall thickness.

Model	(a) (mm)	(b) (mm)	(c) (mm)	(d) (mm)
**Thickness**	2.00	1.96	1.90	1.80

**Table 6:** Models with different friction coefficients.

Model	(a)	(b)	(c)
**Friction coefficient**	0.05	0.10	0.20

**Table 7:** Top expansion length of models.

Model	(a)	(b)	(c)
**Length**	209.831 mm	177.012 mm	157.495 mm

## Data Availability

Data available in a publicly accessible repository.
